# High-Throughput Sequencing-Based Analysis of T Cell Repertoire in Lupus Nephritis

**DOI:** 10.3389/fimmu.2020.01618

**Published:** 2020-08-06

**Authors:** Xiaolan Ye, Zhe Wang, Qiang Ye, Jing Zhang, Ping Huang, Jingying Song, Yiwen Li, Hongjuan Zhang, Feifeng Song, Zixue Xuan, Kejian Wang

**Affiliations:** ^1^Department of Pharmacy, People's Hospital of Hangzhou Medical College, Zhejiang Provincial People's Hospital, Hangzhou, China; ^2^National Engineering Research Center for Protein Drugs, Beijing, China; ^3^GS Medical (Beijing) Technology Development LLC, Beijing, China; ^4^JITRI Applied Adaptome Immunology Institute, Nanjing, China; ^5^Department of Nephrology, Changed Central Hospital, Chengde, China; ^6^Department of Nephrology, People's Hospital of Hangzhou Medical College, Zhejiang Provincial People's Hospital, Hangzhou, China; ^7^Lin He's Academician Workstation of New Medicine and Clinical Translation at The Third Affiliated Hospital, Guangzhou Medical University, Guangzhou, China

**Keywords:** T cell receptor, lupus nephritis, systemic lupus erythematosus, immune repertoire, next-generation sequencing

## Abstract

T cell receptor (TCR)-mediated immune functions are closely related to autoimmune diseases, such as systemic lupus erythematosus (SLE). However, technical challenges used to limit the accurate profiling of TCR diversity in SLE and the characteristics of SLE patients remain largely unknown. In this study, we collected peripheral blood samples from 10 SLE patients with lupus nephritis (LN) who were confirmed by renal biopsy, as well as 10 healthy controls. The TCR repertoire of each sample was assessed by high-throughput sequencing to examine the distinction between SLE subjects and healthy controls. Our results showed statistically significant differences in TCR diversity and usage of TRBV/TRBJ genes between the two groups. A set of signature V–J combinations enabled efficient identification of SLE cases, yielding an area under the curve (AUC) of 0.89 (95% CI: 0.74–1.00). Taken together, our results revealed the potential correlation between the TCR repertoire and SLE status, which may facilitate the development of novel immune biomarkers.

## Introduction

Systemic lupus erythematosus (SLE) is a prototypic autoimmune disorder. As one of the most common and severe complications in SLE, lupus nephritis (LN) is a major cause of SLE-related morbidity and mortality (1, 2). LN requires confirmation by renal biopsy, which is an invasive procedure (3, 4). Without early diagnosis and treatment, LN can usually progress to end-stage renal disease (ESRD) (5). Since it is impractical to perform renal biopsy repeatedly, a non-invasive method for diagnosis and prognosis surveillance of LN is urgently needed (6).

It has been reported that highly diversified T cell receptors (TCRs) are crucial for adaptive immunity in health and disease (7, 8). TCRs are generated by genomic rearrangement of the variable (V), diversity (D), and joining (J) regions, along with palindromic and random nucleotide additions (9). Recently, a series of studies have demonstrated substantial changes in the TCR repertoire of SLE patients (10–13). For instance, Liu et al. (11) found significant differences in V, J, and V–J pairs in SLE patients. And 198 SLE-associated TCR clones were identified for correlation with clinical features (11). However, the changes of TCR repertoire in SLE patients with LN have yet to be described.

In this study, we performed high-throughput sequencing to characterize the TCR repertoire in peripheral blood samples from SLE patients with LN and healthy controls. The results may help understand the property and alteration of T cell immunity in the occurrence and development of SLE.

## Materials and Methods

### Study Participants

A total of 10 SLE patients with LN and 10 healthy controls were recruited from the Zhejiang Provincial People's Hospital, Hangzhou, China. The pathological status of LN patients was confirmed by renal biopsy. The controls were confirmed with no autoimmune disorders or kidney complications. Written informed consents were obtained from all participants. This study was approved by the Ethics Committee of Zhejiang Provincial People's Hospital.

The baseline characteristics of SLE and control groups were presented in Table 1. Following professional guidelines, the diagnosis of LN was confirmed with histopathological examination of renal biopsy. The SLE cases belong to Class-II, Class-IV, and Class-V, respectively. Although the range of age was larger in the SLE group than in the control group (20–68 vs. 35–52), the average age was not significantly different between the two groups (45.9 vs. 45.8). In the current study, we used European League Against Rheumatism (EULAR)/American College of Rheumatology (ACR) classification criteria for SLE, which is a combination of multiple disciplines and international recognition, thus displayed great sensitivity and specificity. The subjects included in our study have multi-organ injury, including hematologic, mucocutaneous, serosal, and renal. In addition, renal biopsy score of class II or V LN is 8 and class II or IV is 10. Therefore, the SLE disease activity index score was 17.40 ± 4.74.

**Table 1:** Basic characteristics of study subjects.

**Basic characteristics**	**SLE group (*n =* 10)**	**Control group (*n =* 10)**
Age (year, mean ± SD)	45.9 ± 16.5	45.8 ± 5.2
Female/Male	9/1	3/7
Low C3 or low C4, No. (%)	9 (90%)	NA
ANA positive, No. (%)	10 (100%)	NA
Anti-dsDNA positive, No. (%)	1 (10%)	NA
Anti-Sm, No. (%)	1 (10%)	NA
Serum creatinine (Scr, μmol/L, the range of normal: 44.0~133)	209.97 ± 277.76	NA
Systemic lupus erythematosus disease activity index score	17.40 ± 4.74	NA
Proteinuria (mg)	1431.35 ± 2076.84	NA
Renal biopsy classification, No. (%)	Class-II: 3 (30%);	NA
	Class-IV: 5 (50%);	NA
	Class-V: 2 (20%)	NA
Clinical domains	Hematologic: 3 (30%)	NA
	Mucocutaneous: 5 (50%)	NA
	Serosal: 3 (30%)	NA
	Renal: 10 (100%)	NA

*Low C3 or low C4, below the lower limit of normal level; ANA, antinuclear antibody; anti-dsDNA, antibodies to double-stranded DNA; anti-Sm, anti-Smith; renal biopsy classification, according to International Society of Nephrology/Renal Pathology Society (ISN/RPS) 2003; Class II, mesangial proliferative lupus nephritis; Class IV, diffuse lupus nephritis; Class V, membranous lupus nephritis*.

### Whole Blood Sample Processing

Peripheral blood mononuclear cells (PBMCs) were extracted from whole blood with Ficoll® to get the highest concentration of lymphocytes. Each type of lymphocyte cell was isolated with monoclonal antibodies specific for the particular lymphocyte cell subset. All cell samples were resuspended in RNAprotect® and stored at 4°C until ready to extract RNA. For low cell counts (<5 ^*^ 10^5^), total RNA was extracted from the Qiagen® RNeasy® Micro kit (catalog #74004). For higher cell counts, RNA was extracted from the Qiagen® RNeasy® Mini kit (catalog #74104).

### Library Construction and Sequencing

RT-PCR multiplex primer sets (iRepertoire, Inc., Huntsville, AL, USA) were used to amplify the CDR3 region of the TCRβ chain. The whole library construction process was automatically operated in the iR-ProcecessorTM and iR-Cassette (iRepertoire, Inc., Huntsville, AL, USA). Then library products with different bar codes were pooled and paired-end sequenced by Illumina MiSeq v2 300-cycle Kit (Illumina Inc.), average read depth of 1M reads each sample (14, 15).

### Raw Data Analysis

Sequences were aligned to TCRβ germline V-, D-, and J-genes according to IMGT/GENE-DB database. Analyzed by the Smith–Waterman algorithm using iR-map pipeline and visualized in iRweb (iRepertoire, Inc., AL, USA). Data analysis included peptide sequences, uCDR3, shared CDR3s, and V- and J-gene usage. Detailed method has been described by Wang et al. (16). The statistics of sequencing quality has been presented in Table S1. The sequencing quality of one SLE sample and one control sample was double-checked and shown in Figure S2. The raw data can be freely downloaded online at: https://figshare.com/search?q=DIO%3A10.6084%2Fm9.figshare.11911284&searchMode=1.

### Statistical Analysis

All statistical analysis was performed using R software (version 3.6.1). Indexes of normal distribution were expressed by mean ± standard deviation. *T-*test for independent samples was performed on comparison between groups. Indexes of non-normal distribution were expressed by median (interquartile interval). Chi-square test was used to compare the counting indexes between groups. Logistic regression was used to analyze the relationship between the specific clone expression level and the clinical outcome. To identify the signature clonotypes, Random Forest analysis (“randomForest” package in R software) together with leave-one-out cross validation was performed to estimate the area under the receiver operating characteristics (ROC) curve and the importance of individual variables.

## Results

### Repertoire Diversity in Systemic Lupus Erythematosus

**Figure 1:**
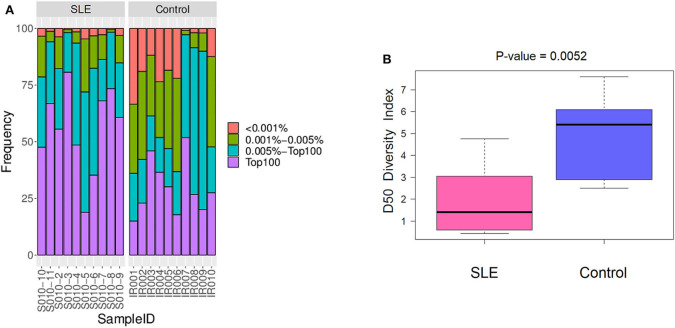
The abundance and diversity of T cell receptor (TCR) clonotype. **(A)** The frequency distribution of different clonotypes. **(B)** The TCR diversity of each group was measured by the D50 index at the level of the V-J combination.

**Figure 2:**
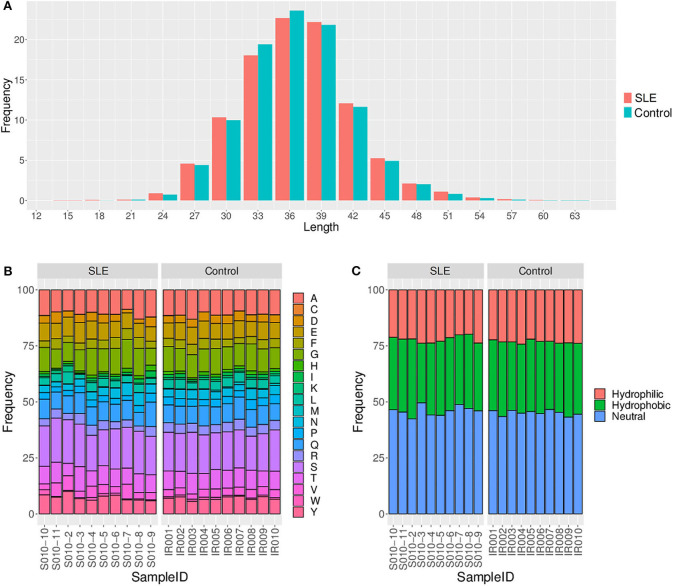
Comparison of the CDR3 length **(A)**, amino acid composition **(B)**, and amino acid hydrophilicity **(C)** between the systemic lupus erythematosus (SLE) group and control group.

We primarily analyzed the abundance and diversity of different TCR clonotypes. The CDR3 sequences were divided into four groups (<0.001, 0.001–0.005, 0.005–top 101, and top 100, respectively) based on their frequency in our samples. The results showed that low abundance clones (i.e., frequency <0.001 and 0.001–0.005%) were less abundant, while top 100 clones were more frequent in SLE individuals (Figure 1A; Table S2), suggesting putatively decreased TCR diversity. The significantly lower D50 diversity index in the SLE group as compared to control group (Figure 1B) further confirmed that the TCR diversity is evidently impaired in SLE. On the other hand, we observed no substantial differences in CDR3 length and amino acid composition between SLE and control groups (Figure 2; Tables S3, S4).

### Characteristics of TRBV and TRBJ Gene Usage in Lupus Nephritis

**Figure 3:**
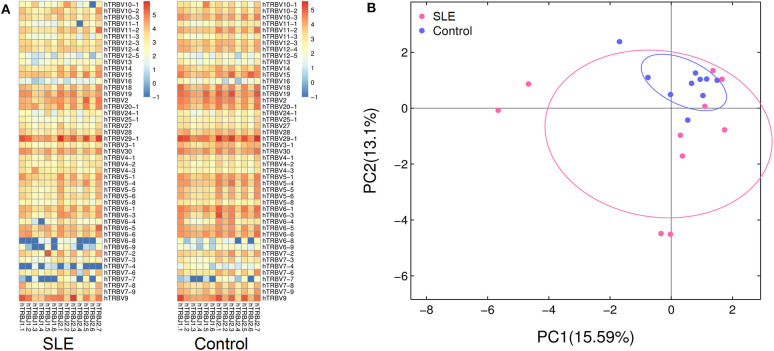
Characterization of TRBV and TRBJ usage. **(A)** The heat map showing frequencies of V-J combinations in the systemic lupus erythematosus (SLE) group and control group. **(B)** Principal component analysis (PCA) based on the abundance of T cell receptor (TCR) clones. The distance between the dots on the graph indicates the degree of dissimilarity of TCR profile between samples.

We then evaluated the gene usage of TRBV and TRBJ in SLE cases and control subjects (Figure 3A). A series of V–J combinations were identified for differential abundance in the two groups (Table 2). We further performed Principal Component Analysis (PCA) on the V–J combination frequency profile. As shown in the PCA plot (Figure 3B), a significant difference was found between SLE and control groups (PERMANOVA *P* < 0.05), as the samples from the control subjects were highly clustered in the upper right quarter of the graph. On the other hand, no obvious difference in sex or renal biopsy classification was detected on the PCA plot (Figure S1).

**Table 2:** V–J combinations with asymmetric expression in the two groups.

**V Gene**	**J Gene**	**Normalized expression in SLE group**	**Normalized expression in control group**	**No. of positive control samples**	**No. of positive SLE samples**
TRBV11-1	TRBJ1-1	88.2 ± 15.6	–	0	3
TRBV12-5	TRBJ2-1	128.6 ± 295.1	–	0	3
TRBV25-1	TRBJ2-3	100.9 ± 8.2	–	0	4
TRBV27	TRBJ1-1	300.7 ± 7.6	–	0	3
TRBV4-1	TRBJ2-5	184.1 ± 41.2	–	0	3
TRBV5-5	TRBJ1-6	56.2 ± 7.2	–	0	3
TRBV6-9	TRBJ2-7	35.9 ± 2.6	–	0	3
TRBV7-6	TRBJ1-5	146.9 ± 24.2	–	0	4
TRBV7-8	TRBJ2-2	163.4 ± 14.0	–	0	3
TRBV10-1	TRBJ1-1	–	58.6 ± 6.6	3	0
TRBV10-3	TRBJ2-6	–	77.4 ± 5.4	5	0
TRBV12-3	TRBJ1-4	–	117.6 ± 3.4	3	0
TRBV12-3	TRBJ2-2	–	567.3 ± 3.7	5	0
TRBV12-4	TRBJ1-4	–	50.4 ± 6.6	3	0
TRBV13	TRBJ2-1	–	43.5 ± 5.3	3	0
TRBV14	TRBJ2-2	–	132.4 ± 5.7	4	0
TRBV28	TRBJ1-6	–	246.1 ± 6.9	3	0
TRBV3-1	TRBJ2-2	–	698.9 ± 2.8	3	0
TRBV3-1	TRBJ2-5	–	34.0 ± 3.5	3	0
TRBV5-5	TRBJ1-4	–	75.1 ± 6.0	3	0
TRBV6-4	TRBJ1-1	–	2163.4 ± 7.3	6	0

*The V–J combinations listed above are widely expressed (n ≥ 3) in one group while not expressed at all in the other group*.

**Figure 4:**
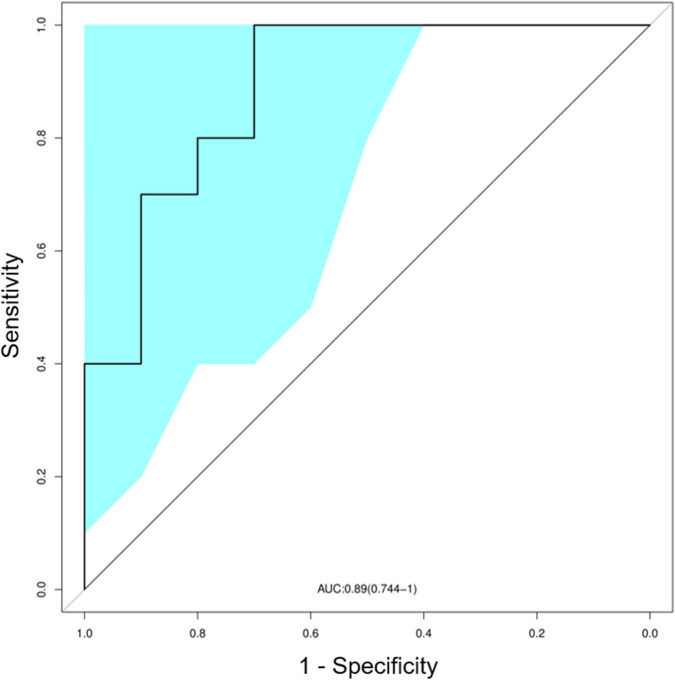
Classification of systemic lupus erythematosus (SLE) by random forest model with a receiver operating characteristics (ROC) curve evaluating the performance. The colored area showed the 95% confidence interval (CI) of the curve.

We also trained a random forest model (see *Materials and Methods*) to evaluate whether the TCR profile could help discriminate between SLE and normal subjects. In the ROC curve, a set of signature clones showed efficient performance in identifying SLE cases. The leave-one-out cross validation yielded an area under the curve (AUC) of 0.89 (95% CI: 0.74–1.00; Figure 4). Such distinction between SLE and control groups promised the possibility of developing TCR biomarkers for early diagnosis of SLE and possibly LN (see *Discussion* below).

## Discussion

To summarize, the results suggested that (1) the SLE status could substantially influence the immune system by impairing the TCR diversity of patients; (2) clear differences in particular V–J combinations could arise between SLE patients and healthy controls; (3) machine learning models were trained to effectively discriminate SLE individuals from control subjects, which may allow the development of diagnostic techniques for early detection of SLE (and possibly LN) risks.

A series of studies have characterized specific signatures of T cell repertoires in patients with various autoimmune diseases (17–19). For instance, Thapa et al. (13) used next-generation sequencing to assess T cell repertoire in peripheral blood (PB) of SLE patients. The results showed a significant decrease in TCR diversity of SLE patients compared to healthy controls (13). In particular, there was evidence that the TCR repertoire profile might serve as a potential biomarker of SLE (11, 12, 20, 21). In addition, Liu et al. (11) reported significant differences in V-J segment usage between the SLE and control groups. However, these studies did not examine the changes of TCR repertoire in LN status. Therefore, some of the differentially expressed clones in our study were not found in previous publications (e.g., TRBV12-5/TRBJ2-1, TRBV6-9/TRBJ2-7, TRBV10-1/TRBJ1-1, TRBV3-1/TRBJ2-2, etc.).

Here we clearly demonstrated that partial expansion of T cells could be observed in SLE patients with LN, which was characterized by decreased TCR diversity and the enrichment or reduction of specific V–J combinations. Due to the altered TCR profile, a series of clonotypes were used as a signature to a trained prediction model. In spite of a limited sample size, our model efficiently discriminated SLE (and possibly LN) individuals from healthy controls, which is worth further validation in larger cohorts. Our pilot study will inspire the subsequent research on the complicated immune environment in SLE and LN.

Of note, our findings are consistent with previously reported results that infiltrating T cells within renal tissue may be targeted toward nephritogenic antigens by the function of TCRβ genes. For example, Massengill et al. (20) found intrarenal lymphocytes in LN showing striking oligoclonal expansion. Our finding also suggested impaired TCR diversity in SLE and possibly LN. Moreover, Sui et al. (12) found the distributions of CDR3, VD indel, and DJ indel lengths to be comparable between the SLE and healthy controls, even though the degree of clonal expansion in the SLE group was significantly greater than in the healthy controls. Likewise, no significant differences in CDR3 length and amino acid composition were detected in our samples. The above evidences corroborated the reliability of our results.

The present study also has several important limitations. First of all, the sample size was relatively small, which impaired the statistical power. Considering potential factors that may confound the TCR characteristics, further studies with larger cohorts and long-term outcome measurements in both SLE patients and matched controls are required to better understand the immunological significance of TCR changes (22, 23). It would be more enlightening if a large sample enables to identify particular LN-specific TCR sequences. Secondly, the current sample did not include those from SLE patients without LN. Since a clinically important biomarker should predict which SLE individuals will develop LN later, subsequent research should make a comparison between SLE patients with and without LN. In addition, the human leukocyte antigen (HLA) gene profiles of the studied subjects are not assessed (24, 25), which may restrict the generalizability of our results.

In summary, we demonstrated a sequencing-based method to present the T cell repertoire characteristics of SLE patients with LN. As T cells play a pivotal role in the etiology of SLE, this study provided a better understanding of TCR-mediated adaptive immunity in SLE. More importantly, our results suggested the potential of developing non-invasive diagnostic solutions for SLE and possibly LN with TCR-based biomarkers.

## Data Availability Statement

All datasets generated for this study are included in the article/Supplementary Material.

## Ethics Statement

The studies involving human participants were reviewed and approved by the Ethics Committee of Zhejiang Provincial People's Hospital. The patients/participants provided their written informed consent to participate in this study. Written informed consent was obtained from the individual(s) for the publication of any potentially identifiable images or data included in this article.

## Author Contributions

ZX and ZW contributed to the conception and design. XY, HZ, YL, JS, and QY acquired the samples. XY, JZ, and FS contributed to the execution of the experiments. PH and KW performed the analysis of the data. ZX, ZW, and KW drafted the manuscript. All authors approved the final version of the manuscript.

## Conflict of Interest

ZW and JZ are employed by the company GS Medical (Beijing) Technology Development LLC. The remaining authors declare that the research was conducted in the absence of any commercial or financial relationships that could be construed as a potential conflict of interest.

## Supplementary Material

Figure S1 Principal component analysis (PCA) plot illustrating sex category **(A)** and renal biopsy classification (class III to class V) of LN patients **(B)**.

Figure S2 As shown in Table S1, subject S010-7, and IR010 seem to be different from other samples with lower rate of reads passing bioinformatics filters. However, removal of these two samples did not lead to substantial changes in the pattern of PCA plot (PERMANOVA *P* = 0.02).

Table S1 Sequence quality control statistics.

Table S2 The frequency of different types of clones for each study subject.

Table S3 The amino acid composition for each study subject.

Table S4 The composition of amino acid hydrophilicity for each study subject.
