# Supplementary figures and images for: High-Throughput Sequencing-Based Analysis of T Cell Repertoire in Lupus Nephritis
Source: Front Immunol. 2020 Aug 6;11:1618. doi: 10.3389/fimmu.2020.01618 (PMC7423971; doi:10.3389/fimmu.2020.01618)

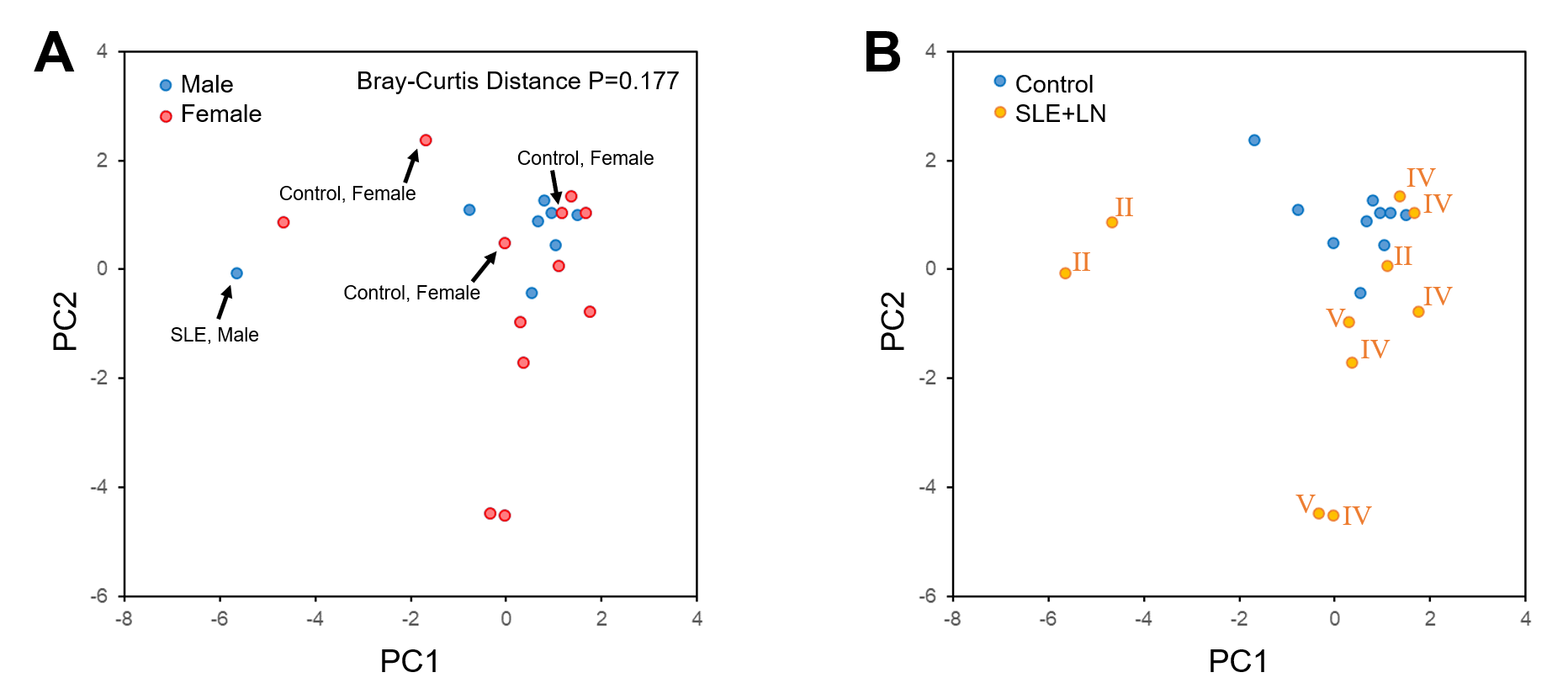

Supplement: Figure S1 — Principal component analysis (PCA) plot illustrating sex category (A) and renal biopsy classification (class III to class V) of LN patients (B). [file Image_1.TIF]

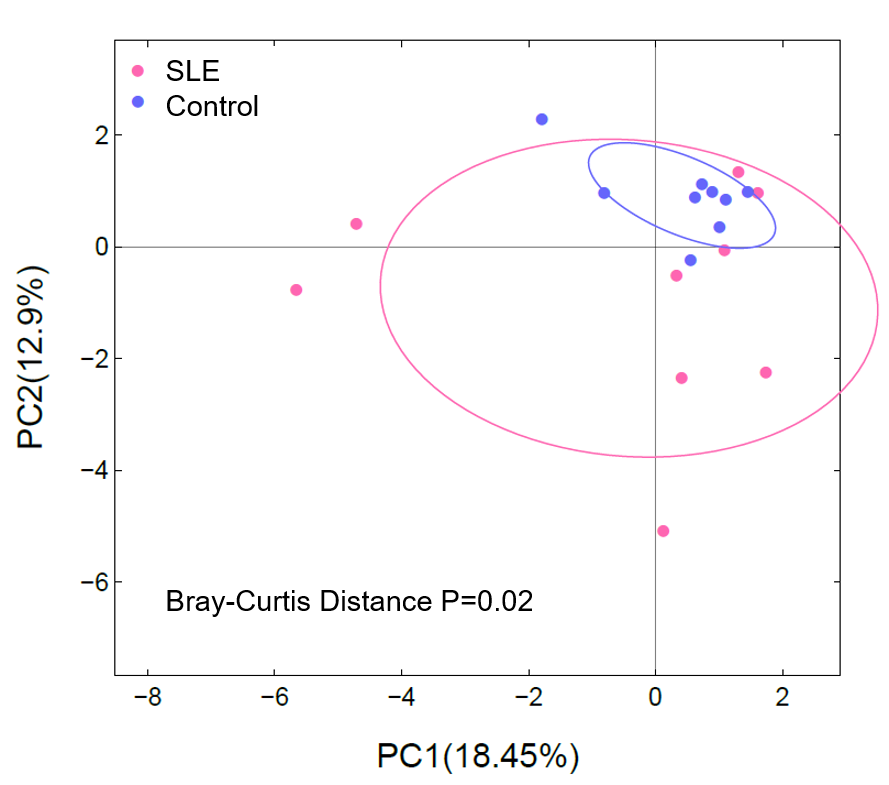

Supplement: Figure S2 — As shown in Table S1, subject S010-7, and IR010 seem to be different from other samples with lower rate of reads passing bioinformatics filters. However, removal of these two samples did not lead to substantial changes in the pattern of PCA plot (PERMANOVA P = 0.02). [file Image_2.TIF]
