# Supplementary material for: High-Throughput Sequencing-Based Analysis of T Cell Repertoire in Lupus Nephritis
Source: Front Immunol. 2020 Aug 6;11:1618. doi: 10.3389/fimmu.2020.01618 (PMC7423971; doi:10.3389/fimmu.2020.01618)
Supplement: Table S1 — Sequence quality control statistics. [file Table_1.DOCX]

Supplementary Material

# Supplementary Tables

**Table S1.** Sequence quality control statistics.

| Group | Sample | Total Demultiplexed Reads | Total Reads Passing Bioinformatics Filters | Passing Rate (%) | Unique CDR3 |
| --- | --- | --- | --- | --- | --- |
| Control | IR001 | 1262669 | 1129136 | 89.42% | 93324 |
| Control | IR002 | 1304273 | 1166548 | 89.44% | 63825 |
| Control | IR003 | 1020815 | 912985 | 89.44% | 39708 |
| Control | IR004 | 990090 | 878454 | 88.72% | 63227 |
| Control | IR005 | 905973 | 811788 | 89.60% | 54850 |
| Control | IR006 | 1216168 | 1085461 | 89.25% | 67859 |
| Control | IR007 | 1181135 | 1062205 | 89.93% | 4392 |
| Control | IR008 | 1485112 | 1319928 | 88.88% | 11331 |
| Control | IR009 | 1178108 | 1055707 | 89.61% | 11629 |
| Control | IR010 | 1154734 | 584539 | 50.62% | 43267 |
| SLE | S010-2 | 1282213 | 887406 | 69.21% | 16594 |
| SLE | S010-3 | 1571263 | 1033545 | 65.78% | 2895 |
| SLE | S010-4 | 2060144 | 1421800 | 69.01% | 8863 |
| SLE | S010-5 | 1132678 | 791704 | 69.90% | 22880 |
| SLE | S010-6 | 1638274 | 1156854 | 70.61% | 16646 |
| SLE | S010-7 | 2385185 | 686452 | 28.78% | 11968 |
| SLE | S010-8 | 1120528 | 735475 | 65.64% | 2690 |
| SLE | S010-9 | 1756552 | 1248948 | 71.10% | 14724 |
| SLE | S010-10 | 969614 | 684157 | 70.56% | 16955 |
| SLE | S010-11 | 2447931 | 1781777 | 72.79% | 7720 |

| **Table S2.** The frequency of different types of clones for each study subject. | | | | | | | | | | | | | | | | | | | | | |
| --- | --- | --- | --- | --- | --- | --- | --- | --- | --- | --- | --- | --- | --- | --- | --- | --- | --- | --- | --- | --- | --- |
| **Category** | **Control Group** | | | | | | | | | | **SLE Group** | | | | | | | | | | **Wilcoxon test P-value** |
|  | **IR001** | **IR002** | **IR003** | **IR004** | **IR005** | **IR006** | **IR007** | **IR008** | **IR009** | **IR010** | **S010-10** | **S010-11** | **S010-2** | **S010-3** | **S010-4** | **S010-5** | **S010-6** | **S010-7** | **S010-8** | **S010-9** |  |
| Top100 | 0.15 | 0.229 | 0.46 | 0.365 | 0.301 | 0.178 | 0.518 | 0.267 | 0.201 | 0.275 | 0.476 | 0.668 | 0.556 | 0.807 | 0.485 | 0.189 | 0.353 | 0.68 | 0.734 | 0.607 | 0.0039 |
| 0.005%-Top100 | 0.211 | 0.193 | 0.154 | 0.153 | 0.169 | 0.19 | 0.454 | 0.648 | 0.699 | 0.202 | 0.311 | 0.272 | 0.267 | 0.175 | 0.45 | 0.531 | 0.471 | 0.183 | 0.249 | 0.241 | 0.31 |
| 0.001%-0.005% | 0.305 | 0.388 | 0.267 | 0.246 | 0.345 | 0.411 | 0.019 | 0.066 | 0.08 | 0.398 | 0.179 | 0.046 | 0.14 | 0.013 | 0.05 | 0.234 | 0.143 | 0.109 | 0.011 | 0.12 | 0.023 |
| <0.001% | 0.334 | 0.19 | 0.119 | 0.235 | 0.184 | 0.221 | 0.009 | 0.019 | 0.021 | 0.125 | 0.035 | 0.013 | 0.037 | 0.006 | 0.015 | 0.047 | 0.033 | 0.028 | 0.006 | 0.032 | 0.026 |

| **Table S3.** The amino acid composition for each study subject. | | | | | | | | | | | | | | | | | | | | | |
| --- | --- | --- | --- | --- | --- | --- | --- | --- | --- | --- | --- | --- | --- | --- | --- | --- | --- | --- | --- | --- | --- |
| **Amino Acid** | **Control Group** | | | | | | | | | | **SLE Group** | | | | | | | | | | **Wilcoxon test P-value** |
|  | **IR001** | **IR002** | **IR003** | **IR004** | **IR005** | **IR006** | **IR007** | **IR008** | **IR009** | **IR010** | **S010-10** | **S010-11** | **S010-2** | **S010-3** | **S010-4** | **S010-5** | **S010-6** | **S010-7** | **S010-8** | **S010-9** |  |
| A | 0.115 | 0.113 | 0.131 | 0.098 | 0.113 | 0.112 | 0.094 | 0.111 | 0.108 | 0.111 | 0.114 | 0.098 | 0.094 | 0.109 | 0.1 | 0.108 | 0.109 | 0.086 | 0.131 | 0.121 | 0.50 |
| C | 0 | 0.001 | 0 | 0 | 0 | 0 | 0 | 0 | 0 | 0 | 0 | 0 | 0 | 0 | 0 | 0 | 0 | 0 | 0 | 0 | 0.34 |
| D | 0.033 | 0.033 | 0.035 | 0.041 | 0.037 | 0.039 | 0.044 | 0.03 | 0.035 | 0.034 | 0.034 | 0.05 | 0.028 | 0.037 | 0.039 | 0.039 | 0.031 | 0.016 | 0.019 | 0.043 | 0.50 |
| E | 0.069 | 0.076 | 0.077 | 0.078 | 0.07 | 0.068 | 0.075 | 0.067 | 0.078 | 0.072 | 0.08 | 0.069 | 0.085 | 0.091 | 0.081 | 0.077 | 0.072 | 0.069 | 0.063 | 0.065 | 0.50 |
| F | 0.037 | 0.039 | 0.046 | 0.046 | 0.042 | 0.034 | 0.026 | 0.034 | 0.042 | 0.041 | 0.029 | 0.046 | 0.029 | 0.031 | 0.042 | 0.039 | 0.049 | 0.049 | 0.048 | 0.031 | 0.86 |
| G | 0.111 | 0.101 | 0.103 | 0.102 | 0.115 | 0.101 | 0.1 | 0.119 | 0.102 | 0.093 | 0.107 | 0.085 | 0.084 | 0.094 | 0.12 | 0.102 | 0.116 | 0.129 | 0.123 | 0.075 | 0.85 |
| H | 0.014 | 0.011 | 0.008 | 0.016 | 0.012 | 0.013 | 0.013 | 0.017 | 0.011 | 0.012 | 0.011 | 0.005 | 0.011 | 0.009 | 0.013 | 0.009 | 0.009 | 0.018 | 0.013 | 0.025 | 0.84 |
| I | 0.014 | 0.018 | 0.009 | 0.01 | 0.012 | 0.015 | 0.011 | 0.012 | 0.016 | 0.012 | 0.011 | 0.005 | 0.009 | 0.003 | 0.013 | 0.012 | 0.012 | 0.01 | 0.019 | 0.029 | 0.81 |
| K | 0.009 | 0.008 | 0.006 | 0.01 | 0.008 | 0.009 | 0.006 | 0.009 | 0.007 | 0.009 | 0.006 | 0.013 | 0.028 | 0.003 | 0.007 | 0.009 | 0.007 | 0.004 | 0.005 | 0.005 | 0.80 |
| L | 0.038 | 0.042 | 0.028 | 0.04 | 0.038 | 0.04 | 0.043 | 0.039 | 0.04 | 0.034 | 0.033 | 0.035 | 0.053 | 0.02 | 0.04 | 0.037 | 0.031 | 0.031 | 0.037 | 0.024 | 0.22 |
| M | 0.003 | 0.003 | 0.002 | 0.003 | 0.007 | 0.005 | 0.011 | 0.004 | 0.003 | 0.01 | 0.002 | 0.002 | 0.002 | 0.001 | 0.002 | 0.004 | 0.003 | 0.001 | 0.001 | 0.001 | 0.012 |
| N | 0.035 | 0.041 | 0.041 | 0.045 | 0.034 | 0.037 | 0.027 | 0.047 | 0.039 | 0.04 | 0.031 | 0.022 | 0.027 | 0.032 | 0.035 | 0.036 | 0.045 | 0.057 | 0.057 | 0.041 | 0.94 |
| P | 0.035 | 0.033 | 0.033 | 0.029 | 0.034 | 0.033 | 0.029 | 0.045 | 0.035 | 0.041 | 0.029 | 0.023 | 0.024 | 0.029 | 0.031 | 0.028 | 0.026 | 0.021 | 0.031 | 0.04 | 0.011 |
| Q | 0.08 | 0.081 | 0.075 | 0.089 | 0.079 | 0.083 | 0.098 | 0.079 | 0.083 | 0.074 | 0.088 | 0.075 | 0.08 | 0.094 | 0.082 | 0.084 | 0.078 | 0.087 | 0.061 | 0.109 | 0.71 |
| R | 0.042 | 0.041 | 0.04 | 0.04 | 0.037 | 0.043 | 0.048 | 0.04 | 0.042 | 0.043 | 0.032 | 0.041 | 0.027 | 0.046 | 0.045 | 0.041 | 0.032 | 0.034 | 0.023 | 0.044 | 0.08 |
| S | 0.173 | 0.169 | 0.184 | 0.173 | 0.164 | 0.171 | 0.17 | 0.152 | 0.166 | 0.184 | 0.181 | 0.197 | 0.195 | 0.19 | 0.159 | 0.161 | 0.18 | 0.182 | 0.19 | 0.172 | 0.07 |
| T | 0.083 | 0.074 | 0.091 | 0.07 | 0.087 | 0.08 | 0.085 | 0.086 | 0.07 | 0.082 | 0.078 | 0.092 | 0.055 | 0.11 | 0.069 | 0.083 | 0.079 | 0.072 | 0.084 | 0.079 | 0.89 |
| V | 0.032 | 0.032 | 0.023 | 0.036 | 0.037 | 0.033 | 0.036 | 0.039 | 0.038 | 0.037 | 0.026 | 0.06 | 0.065 | 0.029 | 0.051 | 0.041 | 0.028 | 0.065 | 0.028 | 0.032 | 0.15 |
| W | 0.007 | 0.009 | 0.01 | 0.011 | 0.01 | 0.006 | 0.004 | 0.006 | 0.007 | 0.007 | 0.022 | 0.003 | 0.004 | 0.004 | 0.012 | 0.011 | 0.01 | 0.004 | 0.005 | 0.004 | 0.92 |
| Y | 0.07 | 0.076 | 0.057 | 0.064 | 0.064 | 0.076 | 0.08 | 0.065 | 0.077 | 0.066 | 0.085 | 0.075 | 0.101 | 0.069 | 0.061 | 0.08 | 0.083 | 0.063 | 0.062 | 0.059 | 0.39 |

| **Table S4.** The composition of amino acid hydrophilicity for each study subject. | | | | | | | | | | | | | | | | | | | | | |
| --- | --- | --- | --- | --- | --- | --- | --- | --- | --- | --- | --- | --- | --- | --- | --- | --- | --- | --- | --- | --- | --- |
| **Category** | **Control Group** | | | | | | | | | | **SLE Group** | | | | | | | | | | **Wilcoxon test P-value** |
|  | **IR001** | **IR002** | **IR003** | **IR004** | **IR005** | **IR006** | **IR007** | **IR008** | **IR009** | **IR010** | **S010-10** | **S010-11** | **S010-2** | **S010-3** | **S010-4** | **S010-5** | **S010-6** | **S010-7** | **S010-8** | **S010-9** |  |
| Hydrophilic | 0.223 | 0.232 | 0.232 | 0.242 | 0.22 | 0.229 | 0.229 | 0.237 | 0.236 | 0.238 | 0.212 | 0.219 | 0.219 | 0.238 | 0.237 | 0.229 | 0.213 | 0.201 | 0.198 | 0.237 | 0.043 |
| Hydrophobic | 0.316 | 0.332 | 0.306 | 0.308 | 0.323 | 0.323 | 0.304 | 0.309 | 0.332 | 0.316 | 0.323 | 0.326 | 0.357 | 0.266 | 0.321 | 0.331 | 0.325 | 0.311 | 0.331 | 0.303 | 0.76 |
| Neutral | 0.461 | 0.435 | 0.462 | 0.449 | 0.457 | 0.447 | 0.466 | 0.453 | 0.432 | 0.446 | 0.465 | 0.455 | 0.425 | 0.496 | 0.442 | 0.44 | 0.462 | 0.488 | 0.471 | 0.46 | 0.23 |
